# Supplementary figures and images for: Primary cilia are present on endothelial cells of the hyaloid vasculature but are not required for the development of the blood-retinal barrier
Source: PLoS One. 2020 Jul 31;15(7):e0225351. doi: 10.1371/journal.pone.0225351 (PMC7394433; doi:10.1371/journal.pone.0225351)

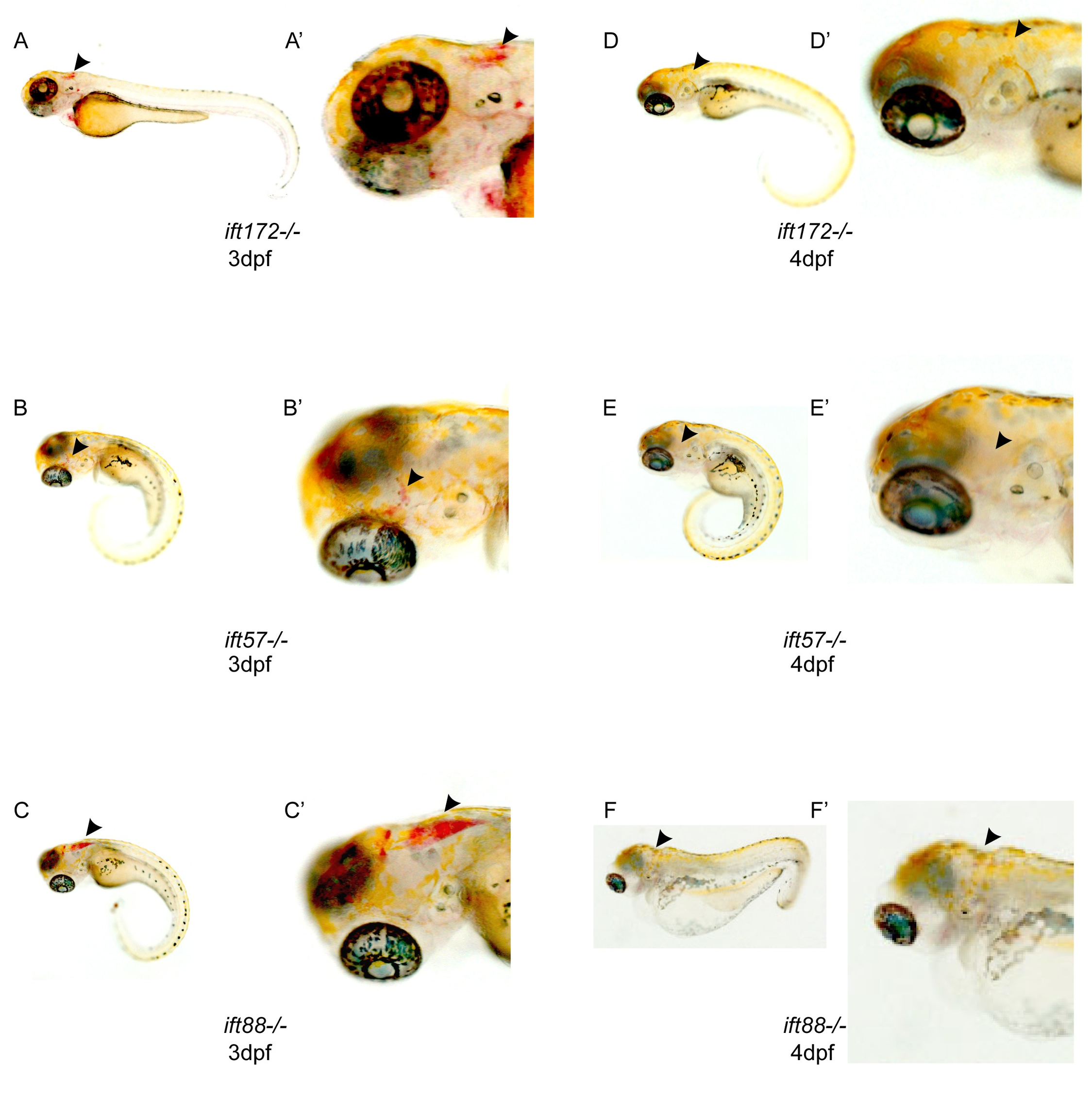

Supplement: S1 Fig — Bright-field images of representative ift172 (A), ift57 (B), and ift88 (C) fish with ICH (arrowheads) at 3 dpf. The same fish were imaged again the following day (4 dpf), and the areas of ICH were largely resolved (D-F, arrowheads). (TIF) [file pone.0225351.s001.tif]
